# Similarity and difference in vegetation structure of three desert shrub communities under the same temperate climate but with different microhabitats

**DOI:** 10.1186/1999-3110-54-59

**Published:** 2013-12-02

**Authors:** Ye Tao, Yuan-Ming Zhang, Alison Downing

**Affiliations:** 1 Xinjiang Institute of Ecology and Geography, Key Laboratory of Biogeography and Bioresource in Arid Land, Chinese Academy of Sciences, South Beijing Road 818, Urumqi, Xinjiang 830011 P.R. China; 2 University of Chinese Academy of Sciences, Yuquan Road 19 (A), Beijing, 100049 P.R. China; 3 Department of Biological Sciences, Macquarie University, Sydney, NSW 2109 Australia

**Keywords:** Species composition, Floristic similarity, Life-form, Nonmetric multidimensional scaling (NMDS), Herbaceous plant, The Gurbantunggut Desert

## Abstract

**Background:**

Community structure and species composition are closely related to plant diversity and ecosystem stability. To explore the similarity in vegetation structure of shrub communities under the same temperate climate but with different microhabitats, 36, 28 and 13 sampling plots in *Ephedra distachya*, *Seriphidium terrae-albae* and *Artemisia songarica* communities were selected respectively, during the course of three seasons (early spring, summer, autumn) in Gurbantunggut Desert, north-western China. The species composition, abundance, biomass and soil nutrients were investigated. Floristic changes were characterized by similarity and ordination methods.

**Results:**

Two communities, *E. distachya* and *S. terrae-albae*, were similar in terms of soil nutrients but differed from the *A. songarica* community*.* Soil organic matter, nitrogen and biological soil crusts accounted for the differences of microhabitats. In spring and summer, more plant families, genera and species were recorded in *E. distachya* and *S. terrae-albae* communities than in the *A. songarica* community but in each community, the number of families, genera, species, herbs and life forms showed a consistent trend summer > spring > autumn. There were significant differences in absolute biomass among the three communities, but the ratio of dead biomass to total biomass was consistently 1:4, indicating the constant turnover rate of plant biomass for nutrient cycling. In each community shrubs accounted for the most biomass. Herbaceous biomass was negligible but the herbs contributed the most richness and abundance.

**Conclusions:**

The similarity in response of all three communities to seasonal changes in vegetation structure and biomass allocation demonstrate convergence although divergence is demonstrated in soil characteristics or microhabitats.

**Electronic supplementary material:**

The online version of this article (doi:10.1186/1999-3110-54-59) contains supplementary material, which is available to authorized users.

## Background

Deserts are ecosystems which take up approximately one fifth of the earth's land surface (Whitford, 2002). Desert ecosystems characteristically lack the biodiversity, stability and levels of soil nutrients found in forest and grassland ecosystems and thus have lower carrying capacity and productivity (biomass) and are slow to recover from disturbance (Rundel and Gibson, 1996; Whitford, 2002; Ward, 2009). Shrubs are a major component of desert vegetation and a key biological resource in the recovery and reconstruction of degraded desert ecosystems where they are of great importance for animal husbandry, soil improvement and as windbreaks for sand stabilisation (Aich, 1991; Li et al., 2006).

Community structure and species composition are the product of evolutionary and environmental drivers that are closely related to plant diversity and ecosystem stability (Putman, 1994; Li, 2000; Eldridge et al., 2007). Plant biomass is an important carrier of material circulation (e.g. C and N cycling), an important step of energy flow (Steinman et al., 1995; Shanmughavel and Francis, 1996) and a key indicator that can be used to evaluate the structure and function of ecosystems (Li et al., 2006). Species composition, diversity and biomass/productivity vary with plant communities and seasons (Stinson and Brown, 1983; Garcia-Pausas et al., 2011; Bischoff et al., 2005; Neves and Oliveira, 2010), but whether there are similar parallel trends (i.e. convergence) in such vegetation variables described above among communities in different microhabitats remains unclear.

The Gurbantunggut Desert is a temperate desert located in the centre of the Junggar Basin, northwestern China. The desert has a relatively higher species richness (Zhang and Li, 1994) and diversity of plant communities (Zhang and Chen, 2002) than other temperate deserts of China. Shrubs such as *Ephedra distachya* L. (Ephedraceae), *Seriphidium terrae-albae* (Krasch.) Poljak. (Asteraceae) and *Artemisia songarica* Schrenk. (Asteraceae), are widely distributed but discontinuous among the sand dunes (Zhang and Chen, 2002). *E. distachya* community is mainly distributed on dunes from the base to mid-dune and *S. terrae-albae* exists at the flat slope; both habitats are commonly covered by biological soil crusts (BSCs). *A. songarica* generally grows either on bare sand or in disturbed situations where there is minimal growth of BSCs.

In recent years, much interest has focused on the species diversity and biomass in the shrub communities of the Gurbantunggut Desert within a single season (Wang et al., 2005; Yu et al., 2010; Song et al., 2011); however, the similarity in vegetation structure of different communities and the seasonal change remain obscure. The three shrub communities above-mentioned chosen in this study were under the same temperate climatic and phenological conditions but with different microhabitats. The objective of this study was to explore the similarity or difference in vegetation structure (including species composition, biomass and community structure) during the course of three seasons (early spring, early summer, early autumn), and to investigate the relationship between vegetation structure and microhabitats. Our hypothesis was that there would be similarity in seasonal changing pattern for vegetation structure but obvious small-scale fluctuation due to microhabitats.

## Methods

### Study area

**Figure 1:**
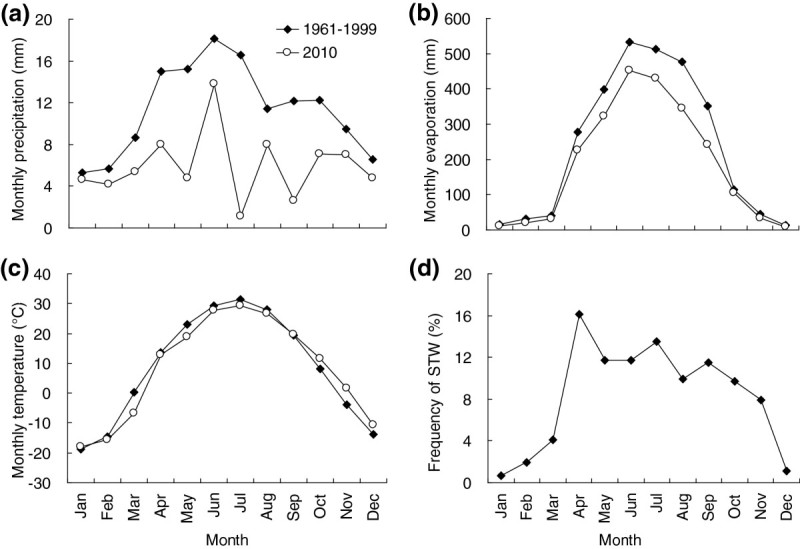
**Monthly changes of precipitation (a), evaporation (b), temperature (c) and frequency of sand transport wind (STW) (d) in the period of 1961 to 1999 and in 2010 at the south area (Caijiahu Town) of Gurbantunggut Desert.**

The Gurbantunggut Desert (44°11′–46°20′ N and 84°31′–90°00′ E) is a typical temperate desert of the world. This is the second largest desert in China with an area of 4.88 × 10^4^ km^2^. The mean annual precipitation is about 70–150 mm, falling predominantly in spring and summer (Figure 1a). In winter, snow accumulates to 10–30 cm. The mean annual pan evaporation is greater than 2,000 mm (Figure 1b). The mean annual temperature is 5–7°C, with the maximum monthly temperature over 30°C and minimum near -20°C (Figure 1c). The trends of monthly evaporation and temperature in 2010 were similar to that in the period of 1961 to 1999 (Figure 1b and c), while the tendency of monthly precipitation was inconsistent with the historical reports, showing very low values in May and July.

The area is covered by massive, dense, semi-fixed sand dunes, from 10 – 50 m in height, running nearly south to north (Qian et al., 2009). Much of the sand surface is covered by BSCs comprising cyanobacteria, lichens and mosses (Zhang et al., 2010b). Winds are predominantly from the WNW, NW and N and wind speeds reach their peak from April to June (Figure 1d), with an annual average of 11.17 m s^–1^ (Wang et al., 2003; Li et al., 2010; Liu et al., 2011b). Eighteen plant communities and 208 vascular plants have been recorded and the natural vegetation is dominated by two small trees, *Haloxylon persicum* Bunge ex Boiss. et Buhse and *Haloxylon ammodendron* (C. A. Mey.) Bunge (Amaranthaceae) (Zhang and Chen, 2002). Additionally, ephemeral and ephemeroid species are both numerous and abundant in desert shrub communities.

### Vegetation sampling

**Figure 2:**
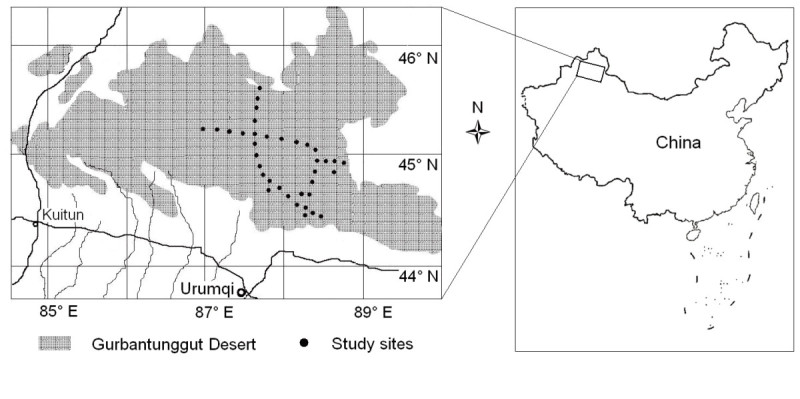
**Study area and collection sites in the Gurbantunggut Desert, north-western China.**

The study sites were located predominately in the south-eastern region of the Gurbantunggut Desert (Figure 2). The study was conducted over three seasons in 2010: early spring (early and mid-April), early summer (late May to early June) and early autumn (mid August to early September). Thirty six sites were randomly selected for *E. distachya* community (EDC), 28 for *S. terrae-albae* (STC) and 13 for *A. songarica* (ASC). The number of sampling sites for each community reflects differences in abundance. The four corners of each plot were marked with wooden stakes and the coordinates, elevation and slope of each plot were recorded. Small seedlings were difficult to identify in early spring so these were marked with small sticks and the plants were identified when more mature in early summer.

Each 20 m × 20 m plot was divided into sixteen 5 m × 5 m sub-plots. In each plot, the shrub species composition and abundance were assessed using the eight 5 m × 5 m sub-plots positioned across the diagonals of the 20 m × 20 m plot. The herbaceous species were surveyed using a 1 m × 1 m area nested within each diagonal 5 m × 5 m sub-plots described above. Following the life form classifications of Zhang and Chen (2002), all recorded species were classified as either: shrub; perennial herbs with long vegetative period (PLV); perennial herbs with short vegetative period (PSV); annuals with long vegetative period (ALV); or annuals with short vegetative period (ASV). However, as PSV and ASV shared the same vegetative period, they were treated as one functional group, that is, as herbs with short vegetative period (HSV). Similarly PLV and ALV were treated together as another functional group, as herbs with long vegetative period (HLV). Nomenclature follows *Flora of China* (ECCAS, 1974–1999) and *Flora Xinjiangensis* (CRFX, 1993–2011).

### Biomass measurement

Biomass of HLV and shrub was determined in autumn. The aboveground (AGB) and belowground (BGB) biomass of HLV were measured at maturity in the eight 1 m^2^ herbaceous sub-plots described above. The AGB of HLV was harvested and the BGB excavated. AGB and BGB were kept and after BGB (roots) was carefully washed, each sample of AGB or BGB was placed into a labelled cloth bag. The biomass samples were oven-dried at 70°C for 48 hours and then weighed on an electronic balance accurate to 0.0001 g.

Five shrubs (*E. distachya*, *S. terrae-albae*, *A. Songarica*, *C. leucocladum* and *Eremosparton songoricum* (Litv.) Vass. (Fabaceae)) were found in all 77 sampling plots. Other than *E. distachya*, the AGB of each shrub was estimated using a general nonlinear allometric equation *Y* = *a CV*^*b*^ (Table 1); where *Y* is AGB, *a* is a normalization constant, *b* is allometric scaling exponent and *CV* is canopy volume. *CV* was estimated as an elliptical cylinder: *C*_L_/2 × *C*_W_/2 × π × height (in centimeter), where *C*_L_ is the crown length (at the widest point, in centimeters) and *C*_W_ the crown width (at the shortest point, in centimeters) (Búrquez et al., 2010). *E. distachya* grows in irregularly shaped clumps so the biomass was determined by the following method. Firstly, in each plot the crown cover of *E. distachya* was measured in eight diagonal 5 m × 5 m sub-plots; secondly, the crown cover of *E. distachya* was measured in three 0.5 m × 0.5 m quadrats, the plants then harvested and dried to determine AGB; AGB for the 8 diagonal sub-plots was calculated using the ratio of biomass to crown cover using the results determined for the three 0.5 m × 0.5 m quadrats.

**Table 1:** **Mathematical coefficients for AGB (** ***Y*** **=** ***a CV***^***b***^**) for four shrubs in this study area**

Shrubs	Coefficients		***n***	***R*** ^2^
	***a***	***b***		
*Seriphidium terrae-albae*	7.98 × 10^–3^	0.817	25	0.907
*Artemisia songarica*	5.23 × 10^–4^	1.011	25	0.840
*Eremosparton songoricum*	4.27 × 10^–7^	2.130	20	0.940
*Calligonum leucocladum*	8.48 × 10^–2^	0.562	20	0.894

All equations are significant at *P* < 0.0001.

The BGB of each shrubs was estimated using root/shoot ratio *R*/*S* (*n* = 5 – 20). The individuals used for *R*/*S* were collected near the sampling plots. *E. distachya* and *E. songoricum* are typical clonal species and it is not possible to identify independent root systems. Thus, five 1 m × 1 m quadrats for *E. distachya* and five 2 m × 2 m for *E. songoricum* (five replicates for each species) were set up and BGB was excavated about 2.0 – 2.5 m deep. *R*/*S* was then calculated by unit area (1 m^2^). All roots of shrubs were washed by running water. All material for biomass determination was oven-dried at 70°C for 72 hours and then weighed using an electronic balance accurate to 0.01 g.

In order to determine dead biomass in autumn, in each plot, eight 0.5 m × 0.5 m quadrats were established in eight diagonal 5 m × 5 m sub-plots to collect dead AGB and BGB. Dead BGB was collected to a soil depth of 50 cm using a corer with an inner diameter of 8 cm. This was gathered using a 0.2 cm sieve in the field and later washed in the laboratory. The live roots were then removed and the dead BGB oven-dried and weighed. Neither dead AGB nor BGB was separated into shrub or herbaceous origins.

### Soil sampling and analyses

Soil properties were only determined in summer as there is minimal seasonal change in soil nutrients in deserts (Gao et al., 2007). In early summer, 2010, a soil sample, consisting of a combined sample from five random points 0–10 cm deep, was collected from each vegetation sampling site. Samples were air dried in the shade after surface organic material and fine roots had been carefully removed. Soil organic matter (SOM), total nitrogen (TN), total phosphorous (TP), total potassium (TK), available nitrogen (AN), pH and electrical conductivity (EC) were determined in the laboratory. SOM was determined by the dichromate oxidation method; pH was measured using a PHS-3C digital pH meter in a 1: 5 soil–water ratio suspension; EC using a DDS-307A conductivity meter (Precision and Scientific Corp. Shanghai, China) in sequence alongside the pH in same suspension; TN by the CuSO4-Se powder diffusion method (GB7848-87); TP by the NaOH fusion-Mo Te Sc colorimetry method (GB7852-87); TK by NaOH fusion-atomic absorption spectrometry (GB7854-87); AN by the alkali hydrolyzation-diffusion method (Bao, 2000).

BSCs were considered to be an important soil factor as BSCs at different developmental levels were broadly distributed throughout the study area (Zhang et al., 2006; Zhang et al., 2010b). The algal and moss crusts were at early and late developmental stages respectively, intermixed with many intermediate stages. Bare sand was recorded as *L*_*i*_ = 0, pure moss crust as *L*_*i*_ = 9, with eight other intermediate stages between bare sand and moss crust. The index of developmental levels of BSCs (IBL) was defined as IBL = Log_10_ (*L*_*i*_ + 1) with a value range of IBL [0, 1].

### Data analyses

Data were analyzed using univariate and multivariate methods. Species richness values for the total number of species in each shrub community were analyzed for each of the three seasons. The species richness values for HSV were calculated in early summer and for HLV in early autumn. Eight soil parameters, species richness values, plant biomass in autumn were compared using one-way ANOVA. Levene’s test was used to test for homogeneity and post-hoc multiple comparisons were performed using Tukey's HSD test. In addition a robust T2 Tamhane’s test was used when variances were not homogeneous (Búrquez et al., 2010). The relationships between soil parameters and species richness/biomass were determined using Pearson Correlation Analysis. SPSS 19.0 statistical package (SPSS Inc. Chicago, Illinois, USA) was used for all the above data analysis.

Similarity coefficients of total species and herb species were analyzed by Sørensen index: *C* = 2*c* / (*a* + *b*), where *C* is the similarity index value, *a*, *b* the numbers of species from communities *A* and *B* respectively and *c* the number of species shared by both communities. The correlations of soil parameters (77 plots × 8 factors) of total species (with the species compositional data) in spring, summer and autumn were done using nonmetric multidimensional scaling (NMDS) analysis through PC-ORD version 5 (MjM Software, Oregon). NMDS is a powerful tool for ecological community data, and it appears to provide a better fit to ecological data than may be obtained using other ordination techniques (McCune and Grace, 2002). The Sørenson (Bray–Curtis) distance with a random starting configuration was used for these analyses. Random starting configurations and 30 runs with real data were used to compute the ordination solution with the dimensionality of each data set assessed by requesting a step down from a six-dimensional ordination. A stable two-dimensional solution was finally identified for all ordinations in all three seasons. We also calculated Pearson correlation coefficients to further elucidate correlations between community patterns and soil parameters in NMDS ordinations. Significance of correlations was determined using the critical values for correlation coefficients (Zar, 1998). Graph of sample plots in ordination space with overlays of environmental variables were used to describe the ordination gradients.

## Results

### Soil characteristics

SOM, TN, AN, EC were comparable in EDC and STC but higher (*P* < 0.05) than those in ASC (Table 2). There was negligible difference in pH values of the three communities (8.18 – 8.41) although the pH of ASC was the highest. TK and IBL were greatly (*P* < 0.05) represented in the order EDC > STC > ASC. TP was highest in STC and least in EDC. Most soil parameters had significant (*P* < 0.05 or 0.01) correlations with the exception of TP. SOM, TN, AN, EC and IBL showed the closest positive relationships; pH was correlated negatively with all other parameters except TP and AN (Table 2).

**Table 2:** **Soil parameters (means ± standard error) and their Pearson correlation coefficients in three communities (EDC, STC and ASC)**

Component	SOM (g kg^–1^)	TN (g kg^–1^)	TP (g kg^–1^)	TK (g kg^–1^)	AN (mg kg^–1^)	pH	EC (μs cm^-1^)	IBL
*Community type* (*n* = 36, 28 and 13 respectively)								
EDC	2.01 ± 0.08a	0.14 ± 0.008a	0.29 ± 0.011b	19.23 ± 1.04a	28.4 ± 2.21a	8.21 ± 0.016a	65.4 ± 1.30a	0.68 ± 0.04a
STC	1.77 ± 0.09a	0.14 ± 0.006a	0.35 ± 0.014a	11.09 ± 0.82b	28.5 ± 1.38a	8.18 ± 0.026a	60.3 ± 3.39a	0.54 ± 0.06b
ASC	1.42 ± 0.05b	0.10 ± 0.003b	0.31 ± 0.012ab	6.69 ± 0.53c	15.8 ± 1.19b	8.41 ± 0.044b	42.0 ± 2.65b	0.25 ± 0.08c
*Soil parameters* (*n* = 77)								
TN	0.820**							
TP	-0.071	0.095						
TK	0.258*	0.108	-0.560**					
AN	0.354**	0.389**	0.042	0.153				
pH	-0.282*	-0.295**	-0.122	-0.296**	-0.188			
EC	0.471**	0.449**	-0.241*	0.523**	0.390**	-0.265*		
IBL	0.519**	0.518**	-0.127	0.456**	0.263*	-0.353**	0.505**	

EDC = *E. distachya* community, STC = *S. terrae-albae* community, ASC = *A. songarica* community. Different letters indicate significant (*P* < 0.05) differences among communities. *indicates significant correlation (*P* < 0.05); **indicates greatly significant correlation (*P* < 0.01).

### Species composition

**Figure 3:**
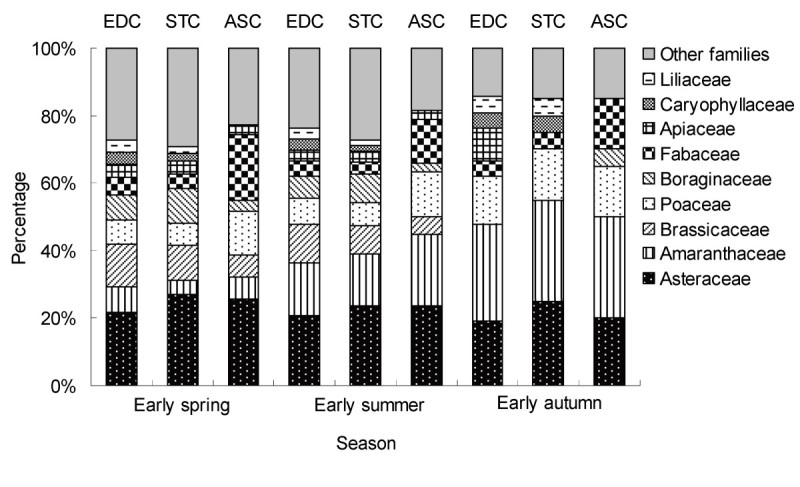
**Family composition of the three communities (EDC, STC and ASC) over three seasons.** EDC = *E. distachya* community, STC = *S. terrae-albae* community, ASC = *A. songarica* community.

A total of 71 species belonging to 64 genera and 24 families was identified in 77 sampling plots during the three seasons; of these, 63, 61 and 40 species, 57, 55 and 37 genera from 23, 23 and 14 families were recorded in EDC, STC and ASC, respectively. The six families with most species, Asteraceae, Amaranthaceae, Brassicaceae, Poaceae, Boraginaceae and Fabaceae, accounted for 61.8% (EDC), 60% (STC) and 69.7% (ASC) of the total number of species in spring and 66.7% (EDC), 63.9% (STC) and 75% (ASC) of the total number in summer respectively (Figure 3). Asteraceae dominated the flora in both spring and in summer, contributing more than 20% of the total number of species. Within each shrub community, the composition of plant families was similar in spring and summer but the number of families, 9 (EDC), 8 (STC) and 7 (ASC), and species, 15, 14 and 16 respectively, declined in autumn.

Results of species similarity indicated that, in spring and summer, the species similarity index values (*C* = 0.857 in spring and *C* = 0.887 in summer) between EDC and STC were more than those between EDC and ASC (*C* = 0.591 and *C* = 0.583), and between STC and ASC (*C* = 0.602 and *C* = 0.554). In autumn, the three community pairs had close to the same similarity index values (*C* = 0.828 – 0.867). In general, the species composition of EDC was similar to STC, but both were obviously richer and more complex than ASC.

### Life-form composition

**Figure 4:**
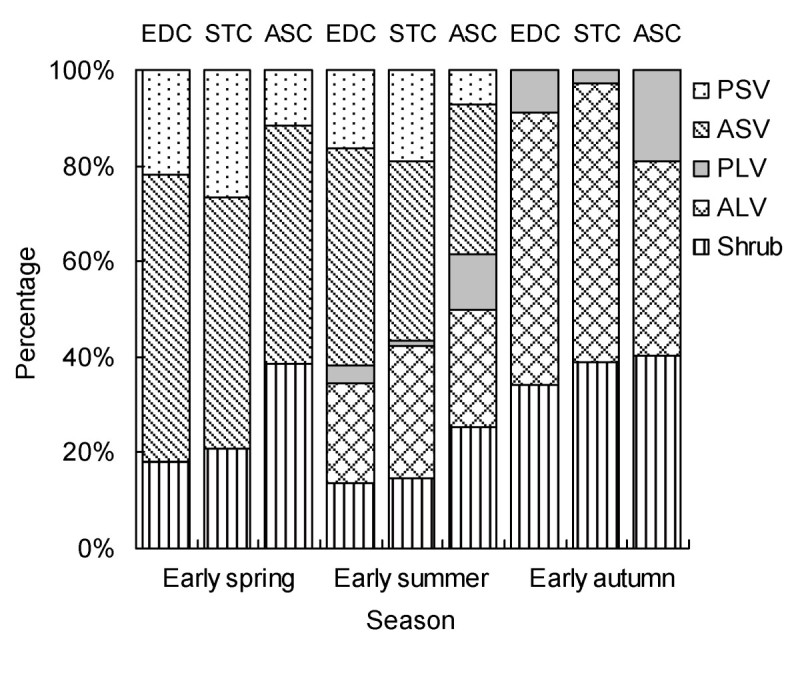
**Composition and relative contribution of five life-forms of the three communities (EDC, STC and ASC) in three seasons.**

The three communities had the same life-form composition within the same season but for each community, the plant life-forms were different in each of the three seasons. There was a similar trend of life-form variation in all communities in the course of the seasons (Figure 4): four life-forms in spring → five in summer → three in autumn. The four life-forms in early spring were shrub, PLV, PSV and ASV. ALV was not present in early spring, but began to appear in later in that season. By autumn, only shrub, PLV and ALV were present. More species of HSV were recorded in spring and summer than in autumn; the percentages in EDC, STC and ASC were 78.04%, 78.21% and 52.01% in spring and 61.76%, 56.57% and 38.52% in summer, respectively. HLV species in the three communities were 24.50%, 28.69% and 36.29% in summer, 66.02%, 60.96% and 59.72% in autumn, respectively. In summary, the life-form composition and ratio to total species number in EDC were similar to STC, but quite different from ASC.

### Species richness

**Figure 5:**
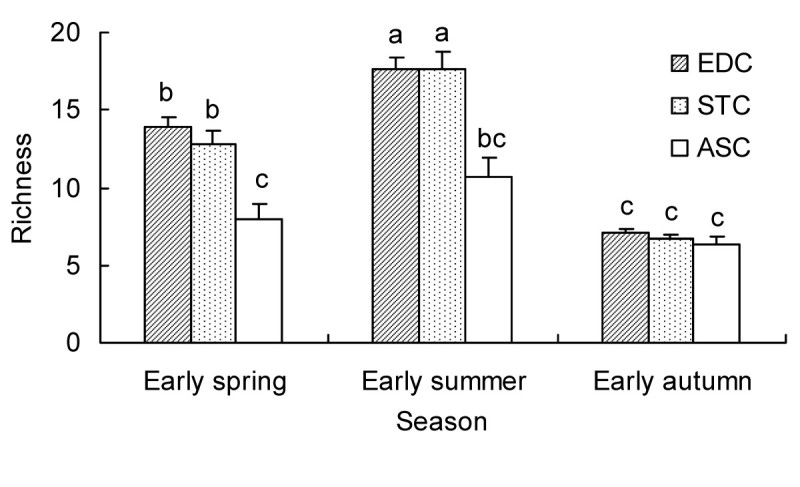
**Species richness of the three communities (EDC, STC and ASC) in three seasons.** Bars represent one standard error. Different letters indicate significant (*P* < 0.05) differences between richness values for each community and season.

There were noticeable seasonal changes in species richness in all three communities. In each community, plant species richness was highest in early summer, followed by spring, with the lowest number of species recorded in autumn (Figure 5). Species richness values for EDC and STC among the three seasons were noticeably different (*P* < 0.05) but there were no significant differences among the three seasons for ASC. Species richness values among the three communities were also different, chiefly in spring (*F* = 10.23, *P* < 0.0001) and summer (*F* = 10.75, *P* < 0.0001). In spring and summer, species richness was similar in EDC and STC, but much more than in ASC. In autumn, species richness values (6.4–7.1) in the three communities were not significantly different (*F* = 1.12, *P* = 0.332).

In early summer, species richness of HSV in each community was the largest among different life-forms, followed by HLV and shrub (Table 3). The mean species richness values of HSV in the three communities were 12.06, 9.25 and 5.54 respectively. In HSV, ASV occupied the larger proportion in each community; in HLV, ALV comprised the larger proportion. Species numbers of other life-forms were different among the three communities with the exception of shrub and PSV. Species numbers of ASV and HSV in EDC were highest, followed by STC, and numbers of both were clearly (*P* < 0.05) higher than those in ASC. Species numbers of ALV and of HLV in STC were evidently higher (*P* < 0.05) than those of EDC and ASC. ASC had the highest species richness of PLV in spite of the mean value of 1.23.

**Table 3:** **Species richness (means ± standard error) of different life-forms (functional groups) in three communities (EDC, STC and ASC) in summer**

Community type	Shrub	HSV			HLV		
		Total	PSV	ASV	Total	PLV	ALV
EDC (*n* = 36)	2.47 ± 0.12a γ	12.06 ± 0.65a	3.36 ± 0.25a β	8.69 ± 0.57a α	3.86 ± 0.26b	0.64 ± 0.14b δ	3.22 ± 0.23b βγ
STC (*n* = 28)	2.54 ± 0.14a β	9.25 ± 0.70a	2.96 ± 0.21a β	6.29 ± 0.65a α	5.07 ± 0.30a	0.18 ± 0.09c γ	4.89 ± 0.26a α
ASC (*n* = 13)	2.57 ± 0.29a α	5.54 ± 0.88b	2.77 ± 0.36a α	2.77 ± 0.68b α	4.00 ± 0.38b	1.23 ± 0.17a β	2.77 ± 0.36b α

Different lowercase Latin letters (a, b and c) and Greek letters (α, β, γ and δ) indicate significant (*P* < 0.05) differences among three communities and four life-forms respectively.

### Biomass allocation

In autumn, total AGB (including dead AGB) followed in the order: STC (125.02 g m^–2^) > EDC (114.01 g m^–2^) > ASC (84.99 g m^–2^) and total live AGB comprised 74.2%, 66.3% and 71.2% of total AGB, respectively (Table 4). Shrubs, in particular the dominant species, accounted for most of the live AGB, while HLV contributed only 5.4%, 5.5% and 5.7% of live AGB in EDC, STC and ASC, respectively. EDC had the largest total BGB (including dead BGB) (69.28 g m^–2^), noticeably more than STC (29.25 g m^–2^) and ASC (23.29 g m^–2^). The total live BGB in the three communities accounted for most of the total BGB, 93.0%, 83.2% and 96.4%, respectively. Similarly, shrubs had the largest live BGB, whereas HLV had the least. There were evident differences between the three communities in TB (including total dead biomass). The total live biomass were 140.05, 117.05 and 82.97 g m^–2^ in EDC, STC and ASC, respectively, most of which was derived from shrubs/dominant species. Live biomass of HLV were 4.75, 9.35 and 4.09 g m^–2^, comprising only 3.4%, 8.0% and 4.9% of total live biomass in EDC, STC and ASC, respectively (Table 4).

**Table 4:** **Above and belowground biomass (means ± standard error) in three communities (EDC, STC and ASC) in autumn**

Component	Community type		
	EDC (***n*** = 36)	STC (***n*** = 28)	ASC (***n*** = 13)
Total AGB (g m^–2^)	114.01 ± 4.19a	125.02 ± 7.04a	84.99 ± 8.31b
Total live	75.65 ± 3.85ab	92.71 ± 7.00a	60.52 ± 7.77b
Shrubs	71.55 ± 3.95ab	84.63 ± 7.20a	57.06 ± 7.98b
Dominant species	67.26 ± 3.96a	74.54 ± 7.03a	51.93 ± 7.37a
HLV	4.09 ± 0.49a	5.06 ± 0.72a	3.47 ± 0.78a
ALV	3.90 ± 0.49a	4.96 ± 0.73a	0.82 ± 0.26b
PLV	0.19 ± 0.07b	0.10 ± 0.04b	2.65 ± 0.75a
Dead	38.36 ± 1.52a	32.30 ± 1.86b	24.47 ± 2.23c
Total BGB (g m^–2^)	69.28 ± 3.66a	29.25 ± 1.50b	23.29 ± 3.40b
Total live	64.40 ± 3.62a	24.34 ± 1.75b	22.45 ± 3.47b
Shrubs	63.75 ± 3.62a	23.08 ± 1.74b	21.82 ± 3.52b
Dominant species	61.51 ± 3.62a	13.84 ± 1.30b	16.94 ± 2.41b
HLV	0.66 ± 0.08a	1.26 ± 0.31a	0.63 ± 0.15a
ALV	0.45 ± 0.06a	0.55 ± 0.08a	0.10 ± 0.03b
PLV	0.21 ± 0.06a	0.71 ± 0.31a	0.53 ± 0.14a
Dead	4.87 ± 0.45a	4.91 ± 0.54a	0.84 ± 0.18b
Total biomass (TB) (g m^–2^)	183.28 ± 7.64a	154.26 ± 8.21b	108.28 ± 11.53c
Total live	140.05 ± 7.44a	117.05 ± 8.56b	82.97 ± 11.00c
Shrubs	135.30 ± 7.55a	107.70 ± 8.75b	78.88 ± 11.29b
Dominant species	128.77 ± 7.59a	88.38 ± 8.33b	68.86 ± 9.78b
HLV	4.75 ± 0.54b	9.35 ± 1.66a	4.09 ± 0.92b
ALV	4.34 ± 0.54a	5.54 ± 0.81a	0.91 ± 0.29b
PLV	0.41 ± 0.11b	3.81 ± 1.56a	3.18 ± 0.90ab
Dead	43.23 ± 1.59a	37.21 ± 1.91b	25.31 ± 2.16c

Different lowercase letters among three communities indicate significant (*P* < 0.05) differences.

**Figure 6:**
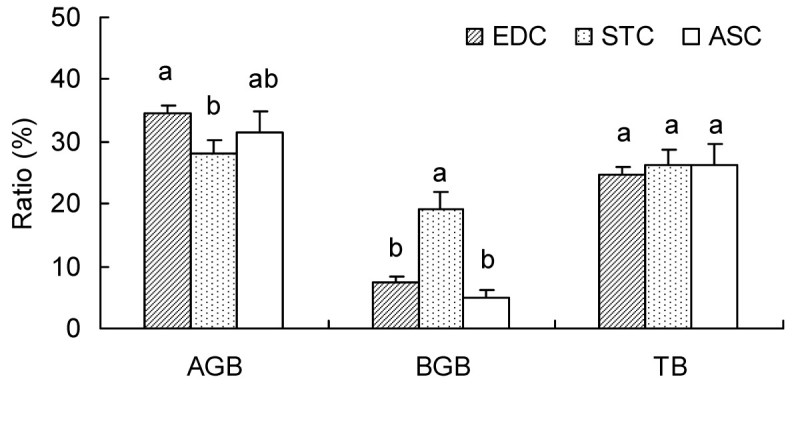
**Ratios of dead biomass to whole biomass (dead + live) in aboveground (AGB), belowground (BGB) and total biomass (TB) of total species in three communities (EDC, STC and ASC) in early autumn.** Bars represent one standard error. Different letters indicate significant (*P* < 0.05) differences between ratios for communities in AGB, BGB or TB.

Although total dead biomass (Table 4), and the ratios of dead AGB to total AGB and dead BGB to total BGB in the three communities (Figure 6) differed considerably, all ratios of total dead biomass to TB were close to 1/4, indicating that in the three desert shrub communities there is a constant turnover rate of plant biomass.

### Relationships between vegetation and soil parameters

Correlations between species richness values and soil parameters were mostly quite low, whereas biomass was considerably influenced by soil parameters (Table 5). pH was negatively correlated with richness of total species in spring and summer, and TK was significantly positive with richness of total species in spring. Species richness of HSV was positively correlated with TK, AN and EC but negatively correlated with pH. There were no significant relationships between any of the soil variables and richness of ALV, or total species in autumn. More than four soil parameters were significantly correlated with most biomass indices but not with AGB of total species. Total live biomass of HSV and ALV and total dead biomass had similar characteristics, both with significantly positive correlations with SOM, TN, AN, EC and IBL, and negative correlation with pH. SOM, TN, TK, EC and IBL were the main factors influencing plant biomass. Thus, the higher levels of soil nutrients produced higher plant productivity/biomass.

**Table 5:** Pearson correlation coefficients between soil variables and species richness components and biomass components across three communities: *indicates significant correlation ( ***P*** < 0.05); **indicates greatly significant correlation ( ***P*** **< 0.01);** ***n*** **= 77**

Component	SOM	TN	TP	TK	AN	pH	EC	IBL
*Richness*								
HSV	0.169	0.200	0.135	0.272*	0.261*	-0.343**	0.280*	0.169
ALV	-0.070	-0.010	0.123	0.135	0.096	-0.124	0.089	0.031
Total species in spring	0.087	0.119	0.179	0.249*	0.209	-0.269*	0.194	0.143
Total species in summer	0.007	0.071	0.207	0.189	0.218	-0.265*	0.184	0.090
Total species in autumn	-0.200	-0.172	0.083	0.112	0.039	-0.004	-0.112	-0.077
*Biomass*								
Total live and dead (TB)	0.393**	0.341**	-0.160	0.328**	0.024	-0.221	0.284*	0.341**
Total live of total species	0.311**	0.255*	-0.165	0.265*	-0.040	-0.141	0.212	0.251*
Total live of HSV	0.400**	0.412**	0.086	0.283*	0.310**	-0.384**	0.408**	0.487**
Total live of ALV	0.330**	0.367**	-0.016	0.117	0.284*	-0.321**	0.431**	0.365**
AGB of total species	0.204	0.236*	0.007	-0.020	-0.060	-0.104	0.094	0.110
BGB of total species	0.325**	0.185	-0.311**	0.509**	-0.001	-0.133	0.276*	0.329**
Total dead	0.423**	0.403**	-0.044	0.325**	0.239*	-0.328**	0.325**	0.414**

**Figure 7:**
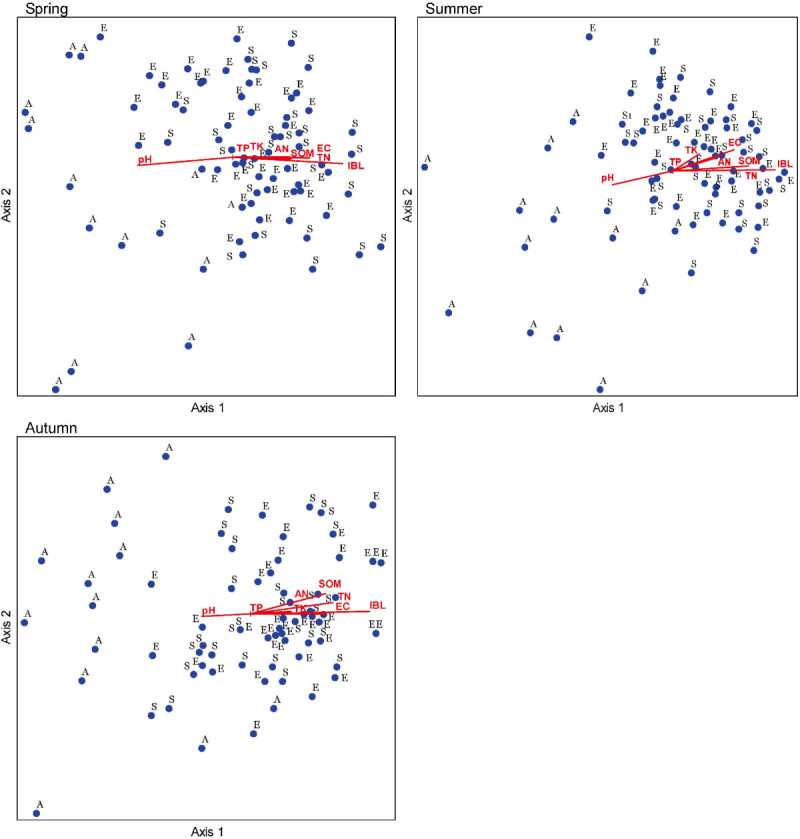
**NMDS ordination diagrams of 77 sampling plots × 8 soil parameters for total species in spring, summer and autumn for the three communities.** E, S and A indicate the plots in *E. distachya* community, *S. terrae-albae* community and *A. songarica* community respectively.

To gain further insight into the relation between soil properties and the three communities, an NMDS ordination analysis was conducted. The cumulative percentage variances explained for the first two NMDS ordination axes were 72.5%, 76.6% and 73.1% in spring, summer and autumn, respectively; and the first axis explained the most proportion of total variation of NMDS ordination in each season (Table 6). Except for TP, all soil parameters were significantly correlated with NMDS axis 1, indicating that the species compositions of three communities were obviously influenced by these soil variables. Noticeably, IBL, TN, SOM and EC showed the highest correlation coefficients (> 0.5) with each NMDS axis 1. NMDS ordination showed an apparent trend that ASC plots clustered separately from EDC and STC plots along axis 1 in each season (Figure 7). From the ordinations, the EDC and STC sampling plots were characterized by higher levels of SOM, TN, IBL, TK and AN (Table 2, Figure 7), whereas ASC had higher pH. Thus each of the three desert communities had a different species composition and different soil properties.

**Table 6:** **Pearson correlation coefficients of soil parameters with first two NMDS ordination axes in three seasons**

Variable	Spring		Summer		Autumn	
	Axis 1	Axis 2	Axis 1	Axis 2	Axis 1	Axis 2
SOM	0.467**	0.115	0.552**	0.145	0.549**	0.287*
TN	0.514**	0.169	0.591**	0.153	0.579**	0.217
TP	-0.008	-0.043	0.044	0.010	-0.068	0.003
TK	0.280**	0.227*	0.349**	0.279*	0.416**	-0.022
AN	0.324**	0.146	0.448**	0.248*	0.406**	0.095
pH	-0.331**	-0.225*	-0.529**	-0.250*	-0.442**	-0.107
EC	0.503**	0.222*	0.553**	0.311**	0.557**	0.037
IBL	0.550**	0.070	0.688**	0.077	0.692**	0.109
Variation explained	42.8%	29.7%	53.8%	22.8%	55.1%	16.2%

*indicates significant correlation (*P* < 0.05); **indicates greatly significant correlation (*P* < 0.01); *n* = 77.

## Discussion

### Similarity and difference in vegetation structure among three desert communities

In this study, species composition and structure differed among three communities, but all had the same trends in seasonal changes. Vegetation is usually closely related to environmental variables and understanding the relationship has been a central issue in plant ecology and synecology (Pausas and Austin, 2001; Jafari et al., 2004; Dias and Melo, 2010). Not only interspecific and intraspecific interactions but also the environmental influence together contribute to the formation and evolution of community structure and function; simultaneously, vegetation effects changes to the environment (Putman, 1994; Li, 2000). In arid areas, water and nutrients are the key factors limiting plant survival (Rundel and Gibson, 1996; Whitford, 2002; Li et al., 2008; Ward, 2009); and in rugged terrain (e.g. hills and sand-dunes), position and aspect have also been shown to be important (Parker, 1988). In the south-western area of the Gurbantunggut Desert which has a stable climate and minor human disturbance (Sun and Yang 2010), heterogeneous microhabitats developed among different communities depending on water content, soil nutrients, soil surface structure and the position on the sand-dune (Qian et al. 2009). Soil water content in layers from 10 cm to 60 cm layers at the bottom of sand-dunes is much higher than soil water content near the top (Wang et al., 2009; Zhang et al., 2010a). EDC and STC mostly grow from the bottom of sand dunes to mid-slope, whereas ASC generally grows in bare sand on the top of sand-dunes. Therefore, we can assume that in the dune system, there is more soil water available to EDC and STC than ASC. It is important also to note that BSCs which are involved in a series of processes which change physical and chemical soil properties, cover the base of dunes but are absent from the top (Zhang et al., 2006; Zhang, 2010b; Zhao et al., 2010). BSCs can improve soil nutrient content (N, P, also K) (Zhang et al., 2006), principally in the 0–5 cm layer. In this study, all eight soil factors are significantly different among the three communities (Table 2). With the exception of pH and TP, the soil parameters for EDC and STC are more than that for ASC. NMDS ordinations and Pearson correlation analysis also reveal that BSC, SOM, TN and EC are most important factors influencing community characteristics and thus the habitat of ASC is very different from those of the other two communities.

We did not investigate the seed banks in this study, but it was obvious from literature that seed banks affected community structure; different habitats also had an effect on seed banks, seed germination and formation of BSCs (Su et al., 2007; Zhao et al., 2010; Liu et al., 2011b). It is difficult for seeds to lodge on the smooth surface of bare sand on dune crests which are also more exposed to wind erosion than the fixed sandy areas at the bottom of dunes or at mid-slope. Seeds are more likely to be blown to the base or mid-slope of sand-dunes, enhancing the resources of the seed bank in those locations (Wang et al., 2003). Wind also reduces the stability of the sandy surface of dune crests making them unsuitable for BSC formation. BSCs can accelerate seed germination, seedling survival, growth and nutrient uptake for most desert species (Long and Li, 2003; Zhang and Nie, 2011), although several studies showed that BSCs inhibited the germination of some species (e.g. *Malcolmia africana* and *Ceratocarpus arenaarius*) (Nie et al., 2009). Larger seed banks can reduce the effect of inhibition by BSCs, augmenting species richness in EDC and STC.

Comparable microhabitats in EDC and STC have produced a complex community structure in EDC and STC with an abundance of species, very different from that found in ASC (Figures 3 and 5; Table 3). In all three communities, the number of life-forms change seasonally from spring (four), to summer (five) and to autumn (three) (Figure 4) and the number of species present in spring and summer exceeds the number present in autumn. Therefore, the similarity in response of all three communities to seasonal changes in vegetation structure (species richness and life-forms) implies convergence; evidence of divergence implicit in response to differences in soil nutrient content, moisture content and BSCs.

### Biomass allocation pattern and nutrient return

The total live biomass of the three communities in the Gurbantunggut Desert in early autumn were relatively low (82.97 – 140.05 g m^–2^). Semi-fixed sand-dunes presented the most well developed landforms (dominated by *Haloxylon* forest) in our study area, with a biomass of 7.53 Mg hm^–2^ (Wang et al., 2005). Therefore, the TB in EDC, STC and ASC accounts for 17.6%, 15.2% and 11.3% of the TB in *Haloxylon* forest, respectively. Dead biomass accounted for a quarter of the total biomass in the three communities. Return of nutrients from plants to the soil was an important step in nutrient recycling, providing resources for further plant growth. In the Gurbantunggut Desert, atmospheric deposition accounts for a small quantity of N (Liu et al., 2011a), but most is derived from the decomposition of dead biomass. The Horqin Sandy Land has higher but varying proportions of dead biomass proportions (40.7% – 65.1%) (Li et al., 2005) than the Gurbantunggut Desert (approximately 25%). In contrast, the proportions in subtropical forest (3.31% – 8.6%) (Shen et al., 2011) and temperate deciduous forest (7.4% – 20.3%) (Fang et al., 2007), are significantly less than those of the desert, owing to the rapid nutrient cycling rate in the forests. The consistent proportions of dead biomass in the three desert communities of this study may suggest a convergent adaptation which enhances the stability of nutrient cycling in the different communities.

Studies have reported that although constituting a relatively small portion (less than 1%) of the total biomass in forest in North America, the herbaceous understory, especially for ephemeral plants, has a quantifiable significance at the ecosystem level, mediating carbon dynamics and energy flow and influencing the cycling rates of essential nutrients, including N, P, K, and Mg (Parsons and Moldenke, 1975; Gilliam, 2007). Despite that the dead biomass was not separated into shrub or herbaceous origins in this study, the herbs accounted for relatively larger proportion of the dead biomass. This indicated that herbs played an important role in soil nutrient cycling in deserts.

Consequently, although there were differences in absolute biomass, biomass allocation patterns on a community-scale among the three desert communities were either the same or very similar, presenting obvious evidence of convergence of desert shrub communities in arid environments.

### Ecological significance of seasonal changes in structure of herbaceous plants

There are numerous herbaceous species in all three communities, and the seasonal changes of herbs represent similarity. The seasonal variations in life-form and species richness are closely related to the appearance and disappearance of herbaceous species (HSV and HLV), especially HSV. Central Asia is considered to the centre of origin and distribution of HSV following the disappearance of the Tethyan Ocean in the Late Neogene and Early Quaternary Period (Mao and Zhang, 1994). Distribution of HSV extended as far as the Mediterranean, Western Asia and North Africa. In China, the Junggar Basin, which includes the Gurbantunggut Desert, is regarded as the easternmost point of HSV distribution (about 205 species) (Mao and Zhang, 1994). In the Gurbantunggut Desert, HSV benefit from limited snow/rainfall in winter and early spring (Mao and Zhang, 1994; Lan and Zhang, 2008) and being to germinate in March and April in spite of low temperatures. HSV flower and mature in May/June prior to the arrival of hot, dry summer conditions.

During the growing period (April to September), the Gurbantunggut experiences very strong winds, predominantly from April to June, in other words, the growth period of HSV (Wang et al., 2003; Li et al., 2010). HSV and BSCs play an important role in reducing sandstorm frequency (Zhang et al., 2006; Zhang et al., 2010b) although sandstorms are less frequent and not as intense as those experienced in other deserts of China (Wang et al., 2003). In early spring, HSV can form dominant synusia or small communities so that some shrub communities can only thrive after HSV has died off in early summer (Zhang and Chen, 2002). Not only does HSV effectively maintain soil surface stability but also generates change in species composition of desert communities (Table 3), important factors in the preservation of biodiversity and stability of desert ecosystems.

Following the demise of HSV in late summer, HLV grew profusely to occupy the eco-niche left by HSV. However, between the disappearance of HSV and appearance of HLV there appears to be an interval during which there is less competition for resources, allowing the two “similar functional groups” to grow at different times during two seasons (summer and autumn). HLV plays an important role in the desert ecosystem until late autumn. The relationship of HSV to HLV was not germane to community type and was a feature shared by all three desert shrub communities.

The total biomass of herbaceous species was exceptionally low in all three communities (Table 4), and the proportions of total biomass of HLV to TB of total species in autumn never exceeded 8.0%. If the study is expanded to a larger scale to include the small tree *Haloxylon*, the proportions of total biomass of HLV to TB of total species in autumn drop to 0.6% (EDC), 1.2% (STC) and 0.55% (ASC). Thus the biomass of herbs is easily overlooked in large-scale estimation of biomass but in the Gurbantunggut Desert the ecological significance (e.g. retaining ecosystem stability and species diversity, preventing sand diffusion, animal husbandry, soil nutrient cycling) of herbs, especially HSV, cannot be disregarded.

## Conclusion

This study represented the similarity and difference in vegetation structure of three desert shrub communities (EDC, STC and ASC) under the same temperate climate but with different microhabitats in the Gurbantunggut Desert, northwestern China. The microhabitats in EDC and STC have produced a complex community structure in EDC and STC with an abundance of species, very different from that found in ASC, but all communities had the same trends in seasonal changes due to the same temperate climate. There were also differences in absolute biomass, biomass allocation patterns on a community-scale among the three desert communities, but there were consistent proportions of dead biomass in the three communities. Thus, the similarity in response of all three communities to seasonal changes in vegetation structure and biomass allocation demonstrate convergence although divergence is demonstrated in soil characteristics or microhabitats.

## Authors’ original submitted files for images

Below are the links to the authors’ original submitted files for images. Authors’ original file for figure 1 Authors’ original file for figure 2 Authors’ original file for figure 3 Authors’ original file for figure 4 Authors’ original file for figure 5 Authors’ original file for figure 6 Authors’ original file for figure 7

## Abbreviations

EDC: *Ephedra distachya* community
HLV: Herbs with long vegetative period
STC: *Seriphidium terrae-albae* community
R/S: Root/shoot ratio
ASC: *Artemisia songarica* community
AGB: Aboveground biomass
ASV: Annuals with short vegetative period
BGB: Belowground biomass
PSV: Perennial herbs with short vegetative period
TB: Total biomass (AGB + BGB)
HSV: Herbs with short vegetative period
BSC: Biological soil crust
ALV: Annuals with long vegetative period
IBL: Index of the developmental levels of BSCs
PLV: Perennial herbs with long vegetative period.
