# Supplementary figures and images for: Similarity and difference in vegetation structure of three desert shrub communities under the same temperate climate but with different microhabitats
Source: Bot Stud. 2013 Dec 2;54:59. doi: 10.1186/1999-3110-54-59 (PMC5432825; doi:10.1186/1999-3110-54-59)

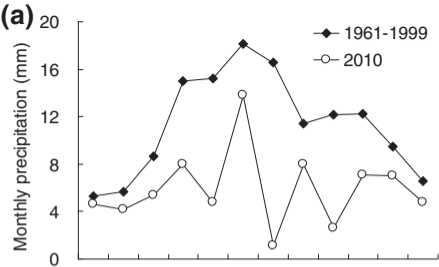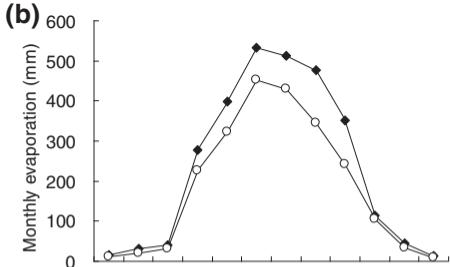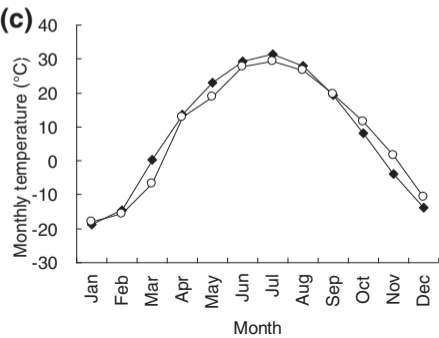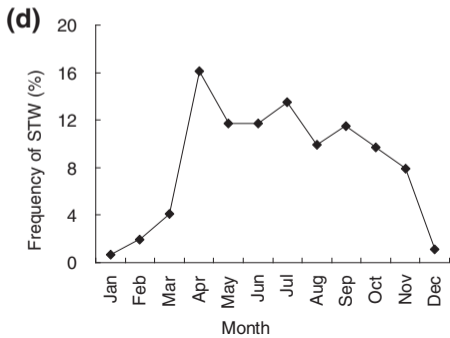

Supplement: Supplementary file 1 — Authors’ original file for figure 1 [file 40529_2012_51_MOESM1_ESM.pdf]

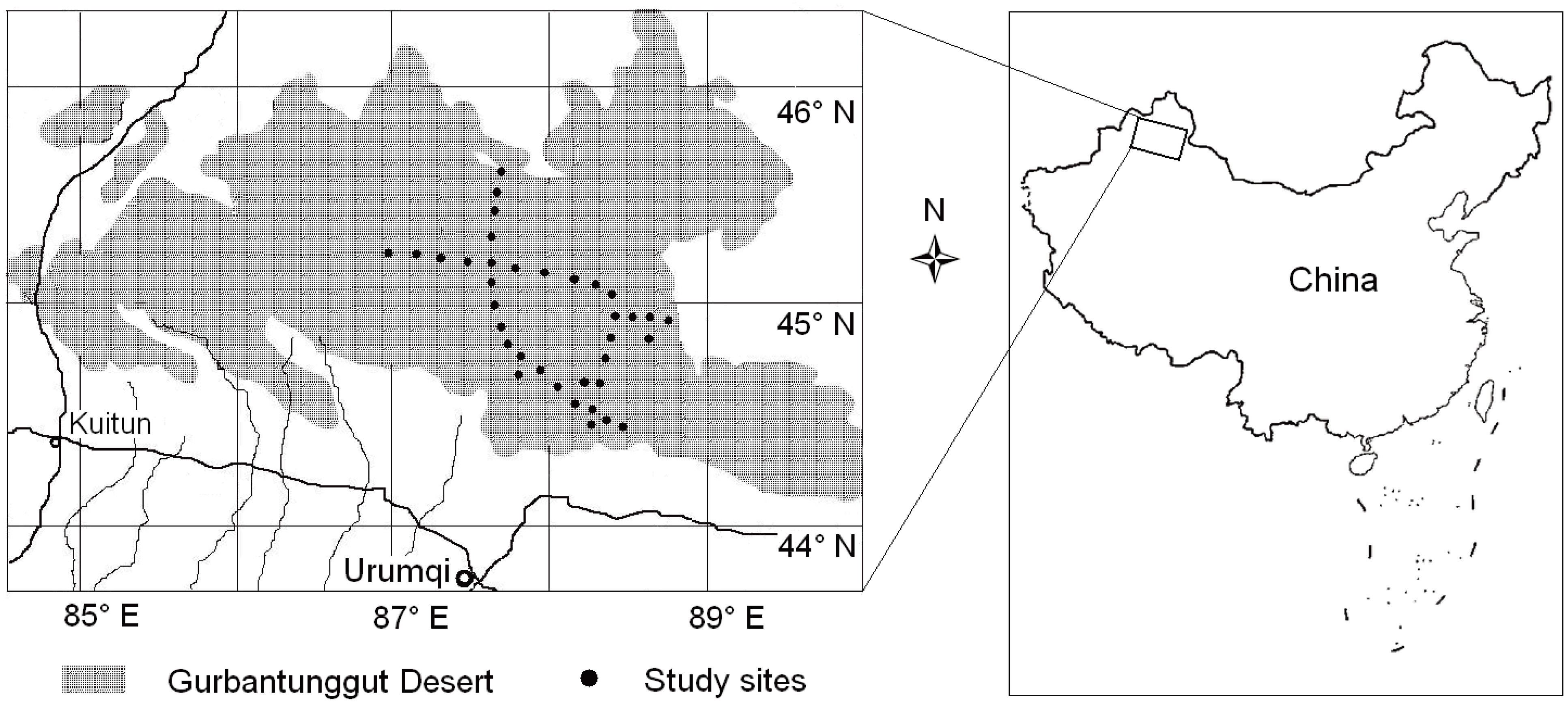

Supplement: Supplementary file 2 — Authors’ original file for figure 2 [file 40529_2012_51_MOESM2_ESM.tiff]

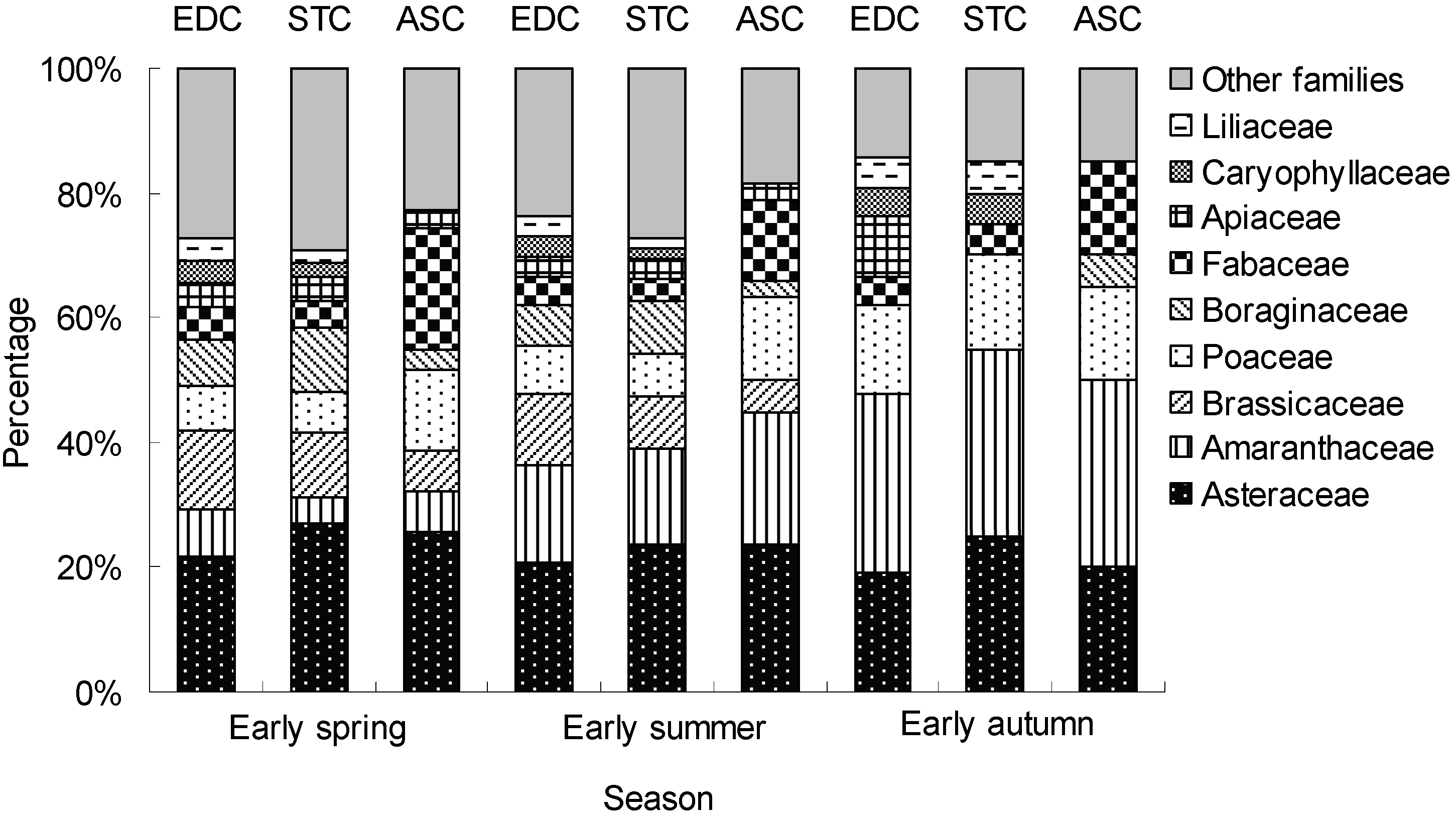

Supplement: Supplementary file 3 — Authors’ original file for figure 3 [file 40529_2012_51_MOESM3_ESM.tiff]

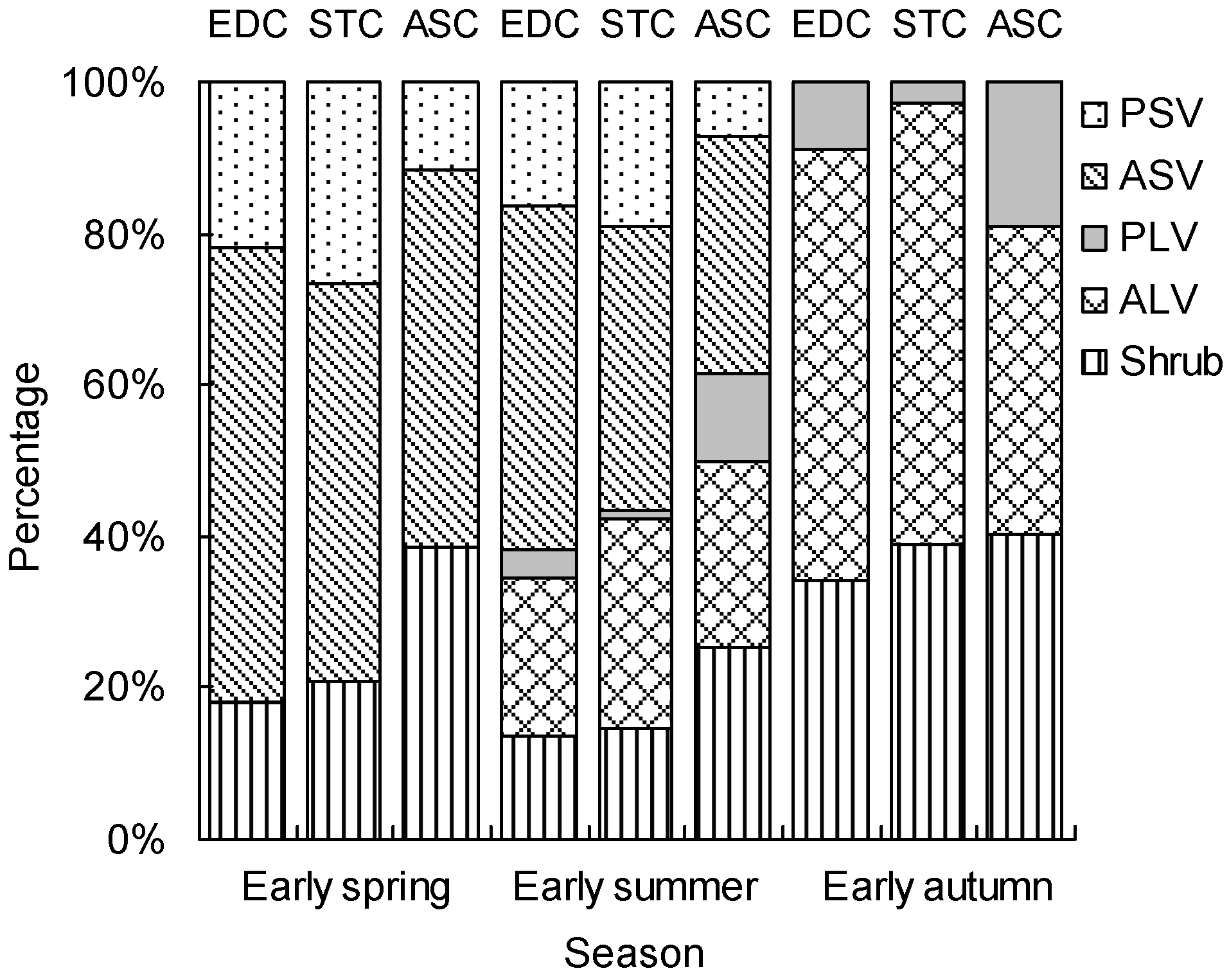

Supplement: Supplementary file 4 — Authors’ original file for figure 4 [file 40529_2012_51_MOESM4_ESM.tiff]

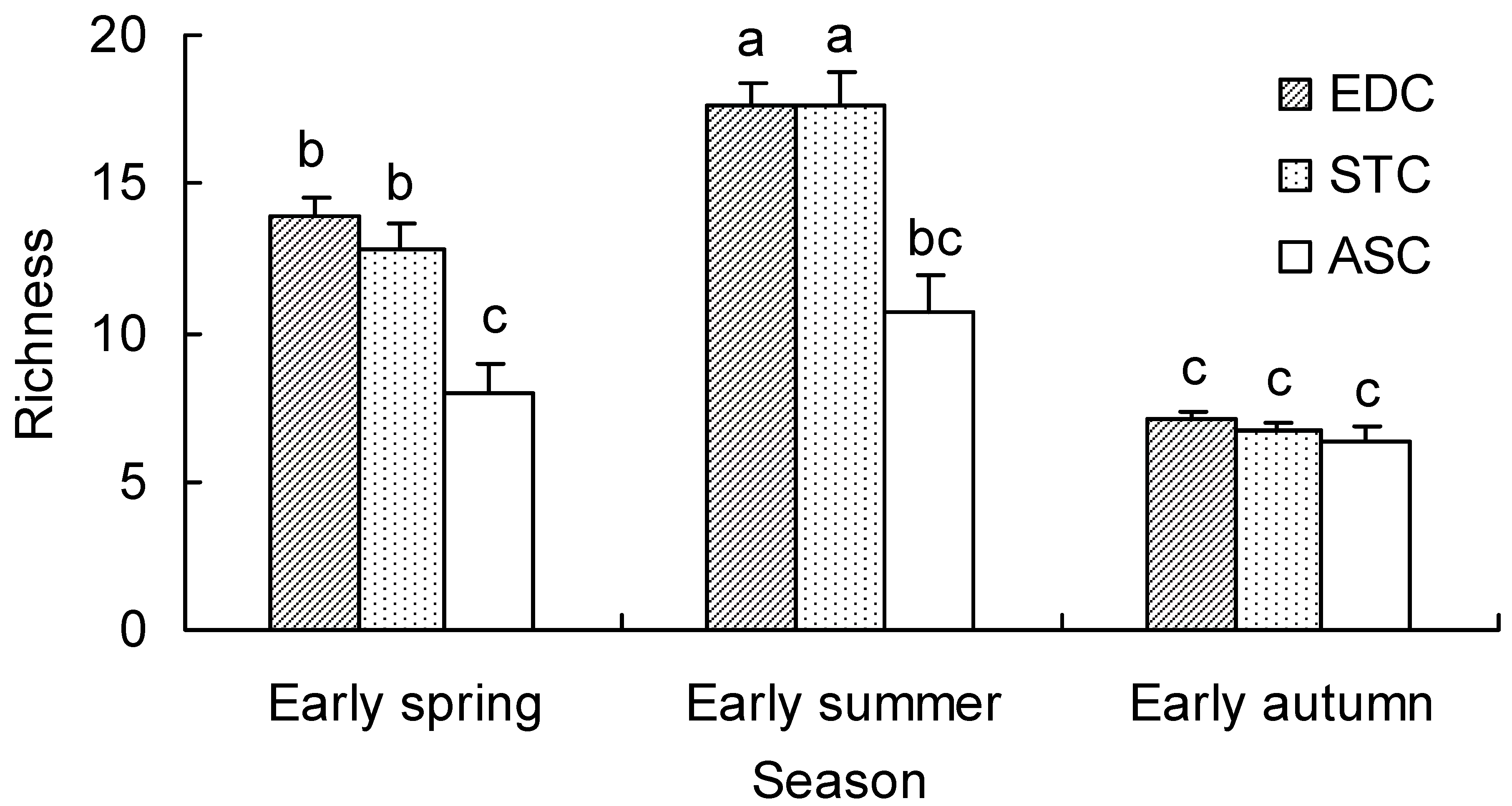

Supplement: Supplementary file 5 — Authors’ original file for figure 5 [file 40529_2012_51_MOESM5_ESM.tiff]

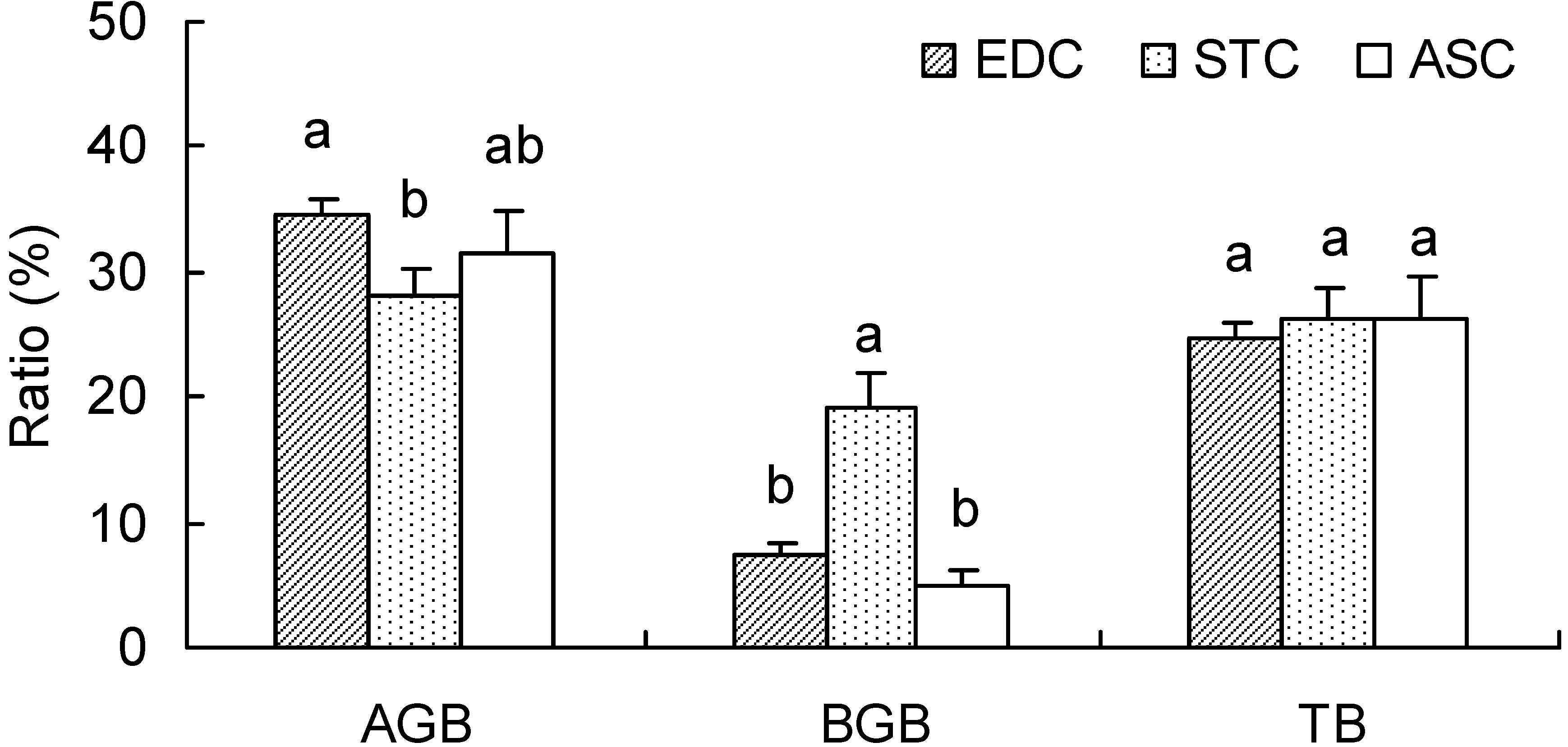

Supplement: Supplementary file 6 — Authors’ original file for figure 6 [file 40529_2012_51_MOESM6_ESM.tiff]

Spring

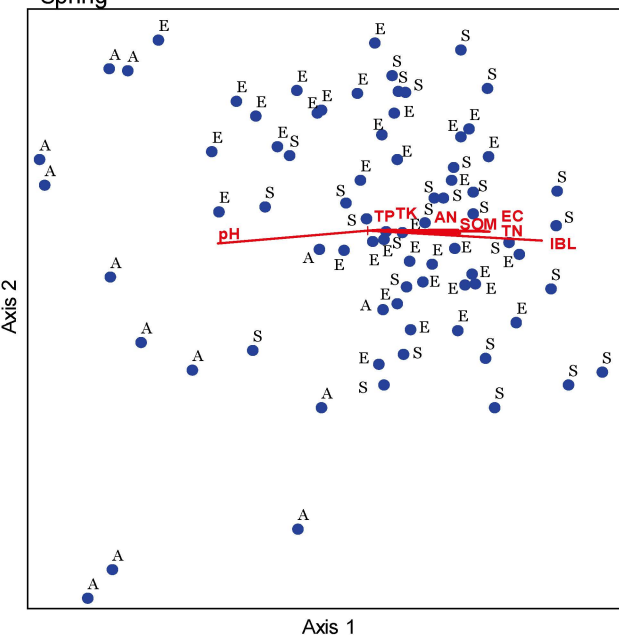

Summer

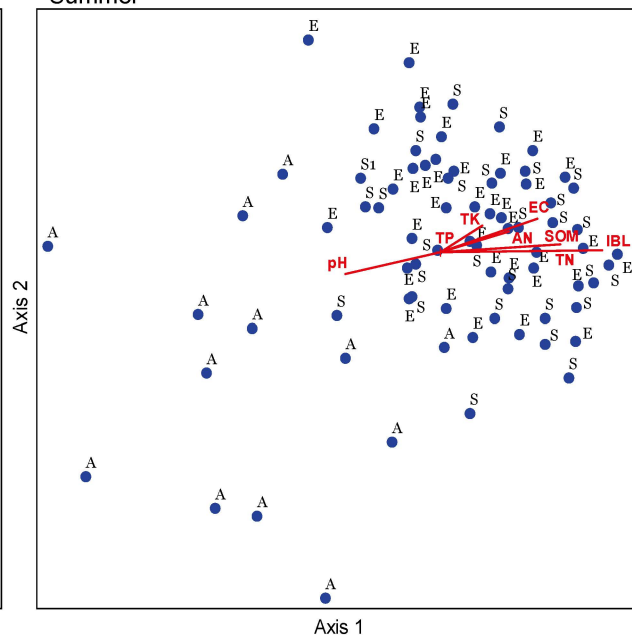

Autumn

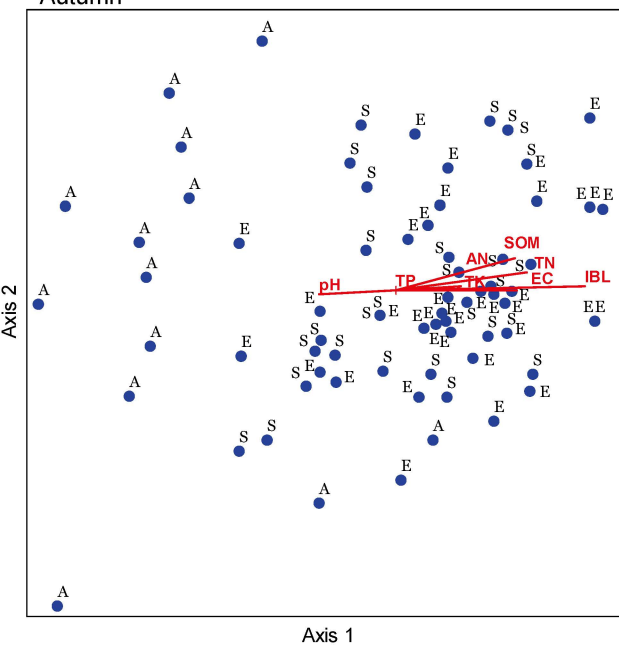

Supplement: Supplementary file 7 — Authors’ original file for figure 7 [file 40529_2012_51_MOESM7_ESM.pdf]
